# Insights into the binding of PARP inhibitors to the catalytic domain of human tankyrase-2

**DOI:** 10.1107/S1399004714017660

**Published:** 2014-09-27

**Authors:** Wei Qiu, Robert Lam, Oleksandr Voytyuk, Vladimir Romanov, Roni Gordon, Simon Gebremeskel, Jakub Vodsedalek, Christine Thompson, Irina Beletskaya, Kevin P. Battaile, Emil F. Pai, Robert Rottapel, Nickolay Y. Chirgadze

**Affiliations:** a Princess Margaret Cancer Center, University Health Network, Toronto, Ontario, Canada; b Hauptman–Woodward Medical Research Institute, IMCA-CAT, Advanced Photon Source, Argonne National Laboratory, Argonne, Illinois USA; c Departments of Biochemistry, Molecular Genetics, and Medical Biophysics, University of Toronto, Toronto, Ontario, Canada; d St Michael’s Hospital, Division of Rheumatology, Departments of Medicine, Immunology and Medical Biophysics, University of Toronto, Toronto, Ontario, Canada; e Department of Pharmacology and Toxicology, University of Toronto, Toronto, Ontario, Canada

**Keywords:** cancer, poly(ADP-ribose) polymerase, TNKS2, structure-based drug discovery, structural biology

## Abstract

The poly(ADP-ribose) polymerase (PARP) family represents a new class of therapeutic targets with diverse potential disease indications. PARP1 and PARP2 inhibitors have been developed for breast and ovarian tumors manifesting double-stranded DNA-repair defects, whereas tankyrase 1 and 2 (TNKS1 and TNKS2, also known as PARP5a and PARP5b, respectively) inhibitors have been developed for tumors with elevated β-catenin activity. As the clinical relevance of PARP inhibitors continues to be actively explored, there is heightened interest in the design of selective inhibitors based on the detailed structural features of how small-molecule inhibitors bind to each of the PARP family members. Here, the high-resolution crystal structures of the human TNKS2 PARP domain in complex with 16 various PARP inhibitors are reported, including the compounds BSI-201, AZD-2281 and ABT-888, which are currently in Phase 2 or 3 clinical trials. These structures provide insight into the inhibitor-binding modes for the tankyrase PARP domain and valuable information to guide the rational design of future tankyrase-specific inhibitors.

## Abstract 2

The high-resolution crystal structures of the human tankyrase 2 poly(ADP-ribose) polymerase (PARP) domain in complex with 16 various PARP inhibitors are reported, including the compounds BSI-201, AZD-2281 and ABT-888, which are currently in Phase 2 or 3 clinical trials.

## 1. Introduction   

The post-translational modification of proteins by poly(ADP-ribosyl)ation is catalyzed by a group of 22 related enzymes which are members of the poly(ADP-ribosylation) polymerase (PARP) family (Schreiber *et al.*, 2006 ▶; Gagné *et al.*, 2006 ▶). The most extensively studied PARP family member is PARP1, which modifies a number of DNA-binding proteins with ADP-ribose chains in response to DNA damage (D’Amours *et al.*, 1999 ▶; de Murcia *et al.*, 1997 ▶; Wang *et al.*, 1997 ▶). Other PARP family members are involved in diverse cellular functions including control of chromatin structure, organization of the mitotic spindle and regulation of signal transduction pathways (Schreiber *et al.*, 2006 ▶).

PARP enzymes catalyze the transfer of an ADP-ribose moiety to aspartate, glutamate, asparagine, arginine or lysine residues of acceptor proteins (reviewed in Hottiger *et al.*, 2010 ▶). The repeating units of ADP-ribose linked by glycosidic bonds can result in polymers that are hundreds of units long, branched and carry a highly polyanionic charge. Poly(ADP-ribose) (PAR) modification is reversible through the action of poly(ADP-ribose) glycohydrolase (PARG; Bonicalzi *et al.*, 2005 ▶), while the final ADP-ribose moiety attached to the protein is removed by ADP-ribosyl protein lyase (Oka *et al.*, 1984 ▶). ADP-ribosylarginine hydrolase-3 (ARH3), an enzyme unrelated to PARG, has also been shown to be capable of PAR hydrolysis (Oka *et al.*, 2006 ▶).

PARP family members share a homologous catalytic domain typically located at the C-terminus of the protein, while the N-terminal sequences contain diverse protein–nucleotide binding or protein-interaction domains. To date, only PARP1, PARP2, PARP3, PARP4, TNKS1 and TNKS2 have been confirmed to be catalytically active (Rouleau *et al.*, 2010 ▶). Common to all active PARP catalytic domains is a conserved signature sequence defined by a ‘catalytic triad’ of histidine, tyrosine and glutamic acid.

Four distinct PAR-binding motifs have been identified: (i) the PAR-binding basic/hydrophobic motif present in DNA-damage checkpoint proteins (Pleschke *et al.*, 2000 ▶) and in heterogeneous nuclear ribonucleoproteins (Gagné *et al.*, 2003 ▶), (ii) the PAR-binding zinc-finger domain (PBZ domain) contained in the CHFR E3 ubiquitin ligase and the DNA-damage response proteins aprataxin and PNK-like factor (APLF; Ahel *et al.*, 2008 ▶), (iii) the mono-ADP-ribose-binding macro domain found in histone H2A (Karras *et al.*, 2000 ▶) and (iv) the WWE domain in RNF146 that recognizes PAR by interacting with iso-ADP-ribose (iso-ADPR) within the poly(ADP-ribose) chain (Wang *et al.*, 2012 ▶). The recognition of ADP-ribose modifications by proteins containing PAR-binding domains can mediate the assembly of multiprotein complexes.

**Figure 1:**
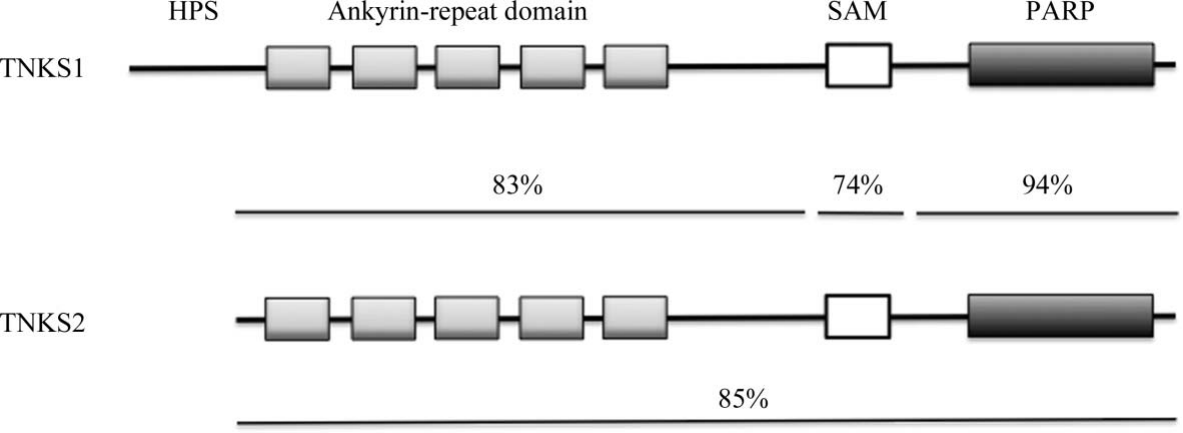
A schematic representation of the TNKS1 and TNKS2 domain architectures and the degrees of sequence identity for the full-length proteins and each domain.

TNKS1 and TNKS2 display a high degree of sequence identity (85% of residues identical overall, with 94% identity in the PARP catalytic domains). TNKS1 and TNKS2 share a common domain organization with a large N-terminal ankyrin domain divided into five ankyrin-repeat clusters (ARCs) involved in substrate recognition, a sterile alpha motif (SAM) domain required for dimerization, followed by the C-terminal PARP domain (Hsiao & Smith, 2008 ▶), as shown in Fig. 1 ▶. TNKS1 contains a unique histidine-, proline- and serine-rich N-terminal region (HPS domain) of unknown function that is not present in TNKS2. TNKS1 was originally identified as a binding partner of the telomerase inhibitor TRF1 and promotes telomere elongation by suppressing the protein expression of TRF1 through an ADP-ribose-dependent ubiquitin pathway (Smith *et al.*, 1998 ▶). Tankyrase enzymes are now appreciated to poly(ADP-ribosyl)ate (PARsylate) a number of target proteins (Hsiao & Smith, 2008 ▶) which contain a common R*XX*P*X*G ARC-binding consensus sequence (Sbodio & Chi, 2002 ▶; Guettler *et al.*, 2011 ▶). TNKS1-deficient cells manifest a cell-cycle defect (Dynek & Smith, 2004 ▶), increased sister-telomere association (Canudas *et al.*, 2007 ▶), spindle dysfunction (Chang *et al.*, 2005 ▶) and altered Glut4/IRAP distribution in adipocytes (Yeh *et al.*, 2007 ▶). TNKS2 has been identified as a binding partner of Grb14 (Lyons *et al.*, 2001 ▶). TNKS2 has also been shown to bind to TRF1 (Hsiao *et al.*, 2006 ▶) and IRAP (Sbodio & Chi, 2002 ▶), suggesting functional redundancy between TNKS1 and TNKS2. While both TNKS1 and TNKS2 knockout mice are viable with a decreased body-weight phenotype (Hsiao *et al.*, 2006 ▶), TNKS1/TNKS2 compound homozygote knockout mice are embryonically lethal by day 9.5, supporting genetic redundancy between the two proteins (Chiang *et al.*, 2008 ▶). Both TNKS and TNKS2 bind to and suppress Axin2, a negative regulator of β-catenin, suggesting that they may represent novel druggable targets for cancers dependent on active β-catenin (Huang *et al.*, 2009 ▶). Loss of TNKS2-dependent negative regulation of the adapter protein 3BP2 underlies the pathogenic mechanism of cherubism, an autosomal dominant disorder affecting cranial bone development (Levaot *et al.*, 2011 ▶). TNKS2 negatively regulates the steady-state levels of the Src-binding adapter protein 3BP2 in macrophages and osteoclasts. Ribosylation of 3BP2 by TNKS2 creates a binding recognition site for the E3-ubiquitin ligase RNF146, which ubiquitylates 3BP2, leading to its destruction by the proteasome (Levaot *et al.*, 2011 ▶). Mutation of the 3BP2 TNKS2 binding site in cherubism patients results in a hypermorphic mutation of 3BP2, leading to its increased expression, activation of Src and hyperactive osteoclasts.

The crystal structures of the catalytic domains of TNKS1 and TNKS2 are highly similar to one another but reveal a number of differences when compared with the catalytic domain of PARP1 (Lehtiö *et al.*, 2008 ▶; Karlberg, Markova *et al.*, 2010 ▶). The nine core β-strands and four α-helices of the TNKS catalytic domain and the histidine, tyrosine and glutamic acid (HYE) triad are conserved in PARP1. However, the N-terminal α-helical domain of PARP1 is entirely absent in TNKS. TNKS1 and TNKS2 also have a much smaller and disordered B-loop than PARP1. This region has been linked to substrate specificity in some mono-ADP-ribosylating enzymes (Karlberg, Markova *et al.*, 2010 ▶). TNKS1 and TNKS2 also differ from other PARP family members in that they harbor a short zinc-binding motif containing residues Thr1079–His1093 within the catalytic domain. Three cysteine residues (Cys1081, Cys1089 and Cys1092) and His1084 coordinate a zinc ion, which is located about 20 Å away from the catalytic site. The function of this substructure is still unknown. A second structural feature which distinguishes the TNKS catalytic domain from PARP1 is that the donor NAD^+^-binding site (D-loop) of TNKS is in a closed configuration compared with PARP1.

The development of inhibitors directed against members of the PARP family has focused mainly on PARP1 and PARP2. Several candidate clinical leads, including BSI-201, AZD-2281 and ABT-888, have progressed to Phase 2 and 3 clinical trials for patients with BRCA mutations in breast or ovarian cancer (Sandhu *et al.*, 2011 ▶; Domagala *et al.*, 2011 ▶; Fogelman *et al.*, 2011 ▶; Liang & Tan, 2010 ▶; Weil & Chen, 2011 ▶). Co-crystal structures of the TNKS catalytic domain with small-molecule ligands (Karlberg, Markova *et al.*, 2010 ▶; Wahlberg *et al.*, 2012 ▶; Narwal *et al.*, 2012 ▶) have provided detailed information about the modes of binding of general PARP inhibitors in comparison to TNKS2-selective inhibitors. Here, we report high-resolution co-crystal structures of the TNKS2 PARP catalytic domain with 16 known PARP inhibitors and provide a consensus structural model for the selectivity of TNKS inhibition distinct from that of PARP1 and PARP2.

## 2. Materials and methods   

### 2.1. Inhibitor compounds   

3-Aminobenzamide (3-AB), 2-(dimethylamino)-*N*-(5,6-dihydro-6-oxophenanthridin-2-yl)acetamide (PJ-34), 5-aminoisoquinolinone (5-AIQ) and 1-piperazineacetamide-4-[1-(6-amino-9*H*-purin-9-yl)-1-deoxy-d-ribofuranuron]-*N*-(2,3-dihydro-1*H*-isoindol-4-yl)-1-one (EB-47) were purchased from Sigma–Aldrich, Calbiochem or Inotek Pharmaceuticals (Beverly, Massachusetts, USA); the rest of the 12 compounds were purchased from other commercial suppliers. Fresh stock solutions of these compounds were prepared in 1% DMSO or distilled water.

### 2.2. Cloning   

A pET-28a vector containing the sequence coding for the PARP domain (*Nde*I/*Xho*I) of TNKS2 was used as a template to generate several PCR fragments of the PARP domain. These PCR fragments were then cloned into pET-28a_LIC (GenBank accession EF442785) and p15TV-L (GenBank accession EF456736) vectors employing a ligation-independent cloning technique (Clontech Laboratories In-Fusion PCR Cloning Kit). Of the eight generated constructs (domain boundaries corresponding to Glu938–Gly1166, Gly950–Gly1162, Ser959–Val1164 and Gly939–Arg1159), one clone (PARP domain boundary Ser959–Val1164 cloned into p15TV_L vector) demonstrated the best expression of soluble protein. This clone, p15TV-L (PARP Ser959–Val1164), was subsequently denoted Tank2.4-6 and was chosen for protein purification.

### 2.3. Protein expression   

Tank2.4-6 DNA was transformed into *Escherichia coli* BL-21(DE3) RIPL cells (Stratagene, La Jolla, California, USA). Cells were grown on standard Terrific Broth (Sigma–Aldrich Canada Co., Oakville, Ontario, Canada) supplemented with 100 mg l^−1^ ampicillin and 34 mg ml^−1^ chloramphenicol in 1 l Tunair flasks at 37°C to an OD_600_ of 3.5; the temperature was then lowered to 16°C and IPTG was added to 0.2 m*M*. Expression was allowed to proceed overnight. The cells were then harvested by centrifugation, flash-frozen in liquid nitrogen and stored at −80°C.

### 2.4. Protein purification   

Cells were thawed on ice and resuspended in binding buffer [100 m*M* HEPES pH 7.5, 500 m*M* NaCl, 5% glycerol, 0.2 m*M* tris(2-carboxyethyl)phosphine, 0.2 m*M* TCEP] supplemented with 0.5% CHAPS, 0.25 m*M* phenylmethylsulfonylfluoride and 0.5 m*M* benzamidine. After disruption by sonication and centrifugation at 60 000*g* for 40 min, the cell-free extracts were passed through a DE-52 column (5 cm diameter × 7.5 cm) which had been pre-equilibrated with the same buffer and were then loaded by gravity flow onto a 10 ml Ni–nitrilotri­acetic acid (NTA) column (Qiagen, Germantown, Maryland, USA). The column was washed with five column volumes (CV) of wash buffer (100 m*M* HEPES pH 7.5, 500 m*M* NaCl, 5% glycerol, 15 m*M* imidazole, 0.2 m*M* TCEP) supplemented with 0.5% CHAPS, followed by five volumes of wash buffer. The His_6_-tagged protein was eluted with the same buffer containing 250 m*M* imidazole. This sample was concentrated using a Vivaspin unit (Sartorius NA, Edgewood, New York. USA) and loaded onto a 2.6 cm diameter × 60 cm Superdex 200 column (GE Healthcare) equilibrated with gel-filtration buffer (10 m*M* HEPES pH 7.5, 500 m*M* NaCl, 0.2 m*M* TCEP). Elution was carried out at a flow rate of 3 ml min^−1^ at 8°C and Tank2.4-6 was eluted as an apparent monomer. This sample was concentrated to ∼1 ml, diluted tenfold with ion-exchange buffer (20 m*M* MES buffer pH 6.5, 5% glycerol, 0.2 m*M* TCEP) and subjected to cation-exchange chromatography on a 1.6 cm diameter × 10 cm Source 30S column (GE Healthcare). The column was washed with 3 CV of 50 m*M* NaCl in the same buffer and developed with a 20 CV linear gradient of NaCl (50–500 m*M*). Tank2.4-6 eluted at ∼375 m*M* NaCl. It was immediately concentrated to 25 mg ml^−1^, divided into 1.25 mg aliquots, flash-frozen and stored at −80°C.

### 2.5. *In vitro* PARP assay   

**Figure 2:**
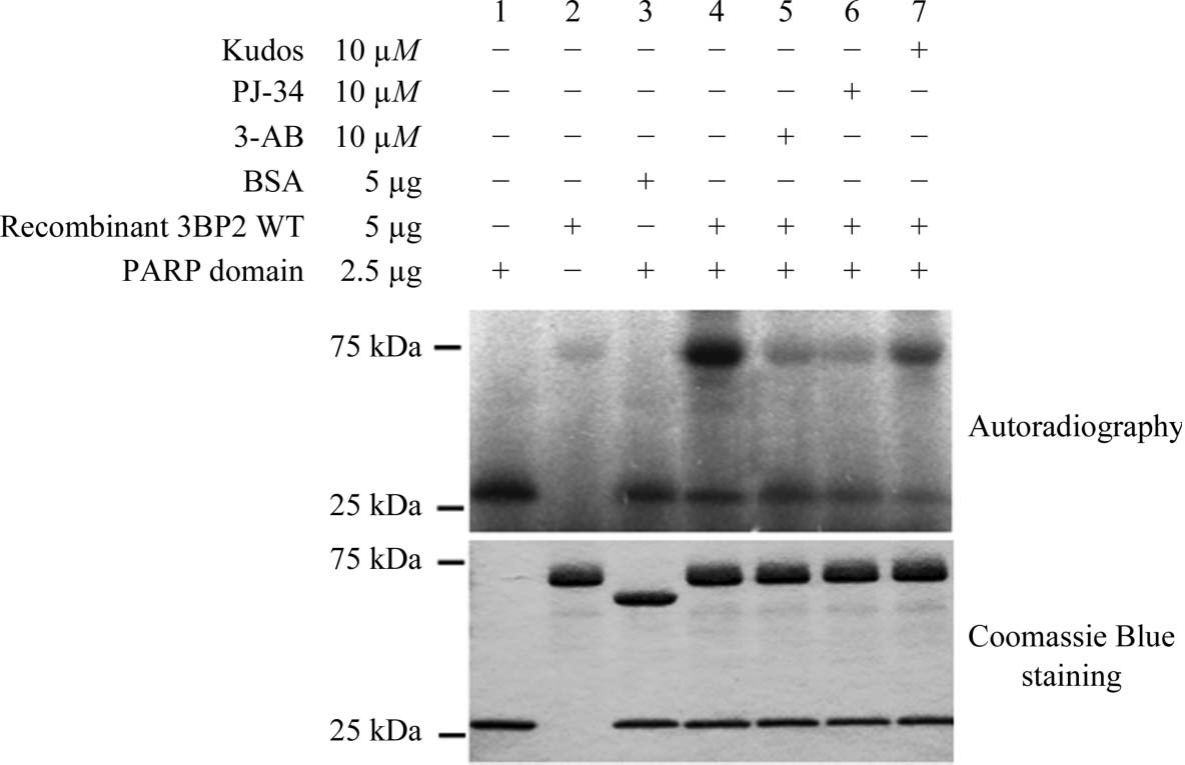
Purified PARP domain of TNKS2 is catalytically active and competent to ribosylate recombinant 3BP2 protein *in vitro*. An *in vitro* PARsylation assay was performed using purified PARP domain of TNKS2 and 3BP2 (lanes 4–7) as a substrate or BSA (lane 3) as a control. The PARP inhibitors 3-AB (lane 5), PJ-34 (lane 6) and AZD-2281 (lane 7) were used to inhibit the activity of the PARP domain. Reactions with PARP domain (lane 1) or 3BP2 (lane 2) alone were performed as negative controls. The amount of purified PARP domain and 3BP2/BSA used for reaction was confirmed by Coomassie Blue staining (lower panel).

Purified PARP domain of TNKS2 and either BSA or recombinant full-length 3BP2 protein were incubated in PARP reaction buffer (50 m*M* Tris pH 8.0, 4 m*M* MgCl_2_, 0.2 m*M* dithiothreitol) containing 0.5 m*M* NAD^+^ as an exogenous source of ADP-ribose for 30 min at 25°C with or without PARP inhibitors. Reactions were stopped by adding sample buffer to the tubes. Samples were boiled and separated on a 4–20% SDS–PAGE gel. The gel was stained with Coomassie Blue, dried on a gel dryer and used for autoradiography analysis (Fig. 2 ▶).

### 2.6. Crystallization   

The TNKS2 protein sample was prepared at a concentration of 15 mg ml^−1^ (0.06 m*M*) and incubated with 0.1 m*M* inhibitor for 1 h. 1.0 µl of the mixture was then transferred to a hanging drop and mixed with an equal volume of reservoir solution consisting of 0.2 *M* NaCl, 0.1 *M* HEPES buffer pH 7.5, 12–15% isopropanol. The rod-shaped crystals were fully grown after one week to standard dimensions of 100 × 30 × 30 µm. In co-crystallization experiments, the crystals were mounted and transferred into a droplet that contained identical components to the actual drop on the crystallization plate plus 0.1 m*M* of the respective inhibitor and 10% glycerol. Using a ‘co-crystallization plus soaking’ technique, before introducing the cryoprotectant the crystals were soaked overnight in 10 m*M* inhibitor. An equal amount of inhibitor (10 m*M*) and 10% glycerol were added to the cryoprotectant. In ‘inhibitor replacement’ experiments, the crystals were grown in the presence of 3-AB (the crystals were easy to reproduce and 3-AB has a relatively low affinity for TNKS2 when compared with the other inhibitors) and then replaced with the inhibitor of interest. In this approach, crystals were grown at room temperature with 0.1 m*M* 3-AB under the conditions described above. Prior to harvesting, crystals were soaked overnight with 5–10 m*M* of the respective replacement inhibitor. The cryoprotectant solution included 5–10 m*M* of the replace­ment inhibitor and 10% glycerol. Cryoprotected crystals were flash-cooled in liquid nitrogen for low-temperature X-ray screening and data collection.

### 2.7. X-ray data collection and processing   

Synchrotron X-ray data sets for TNKS2 inhibitor complexes were collected at 100 K on beamlines 17-ID and 17-BM at the Advanced Photon Source, Argonne National Laboratory. In-house data sets were collected on a Rigaku FR-E Super­Bright rotating-anode generator equipped with a Rigaku Saturn A200 CCD detector (Rigaku, The Woodlands, Texas, USA). The diffraction data were reduced and scaled with *XDS* (Kabsch, 2010 ▶).

### 2.8. Structure determination and crystallographic refinement   

The crystals of all complexes belonged to space group *P*2_1_2_1_2_1_, with unit-cell parameters around *a* = 74, *b* = 79, *c* = 153 Å and four molecules per asymmetric unit. The first complex crystal structure was determined by molecular replacement with *MOLREP* (Vagin & Teplyakov, 2010 ▶) using TNKS1 (PDB entry 2rf5; Lehtiö *et al.*, 2008 ▶) as a search model. The rest of the complex structures were determined by the difference Fourier method. Following the initial rigid-body refinement, interactive cycles of model building and refinement were carried out using *Coot* (Emsley *et al.*, 2010 ▶) and *BUSTER-TNT* (Bricogne *et al.*, 2011 ▶). The coordinates and topologies of the ligands from this study were generated using the GlycoBioChem *PRODRG*2 server (Schüttelkopf & van Aalten, 2004 ▶). Ligands were introduced at the last stages of refinement after most of the protein models of TNKS2 has been built. Water molecules as well as other solvent ligands were added based on the 2*mF*_o_ − *DF*_c_ map in *Coot* and were refined with *BUSTER-TNT*. Using *phenix.refine* (Afonine *et al.*, 2012 ▶), a simulated-annealing map based on the final model without any inhibitors and waters was generated for each complex structure as a reference to avoid model bias. Owing to the crystal packing, the inhibitor electron density had different quality for each of the four TNKS2 molecules in the asymmetric unit, among which chain *D* had the worst density in most complexes, while chains *A*, *B* and *C* had equally high-quality electron density. In order to have a direct comparison, we choose chain *C* in our discussion below except for the situations where the chain is specifically mentioned. In the case of the BSI-201 complex structure, an additional experimental phasing map was generated using *phenix.autosol* (Adams *et al.*, 2010 ▶), which proved that there were ten iodine sites per asymmetric unit in the complex structure and that they corresponded to the ten BSI-201 positions in the final model. The refinement statistics are listed in Table 1 ▶. All figures except for Figs. 1 and 2 were produced using *PyMOL* (http://www.pymol.org).

## 3. Results   

**Figure 3:**
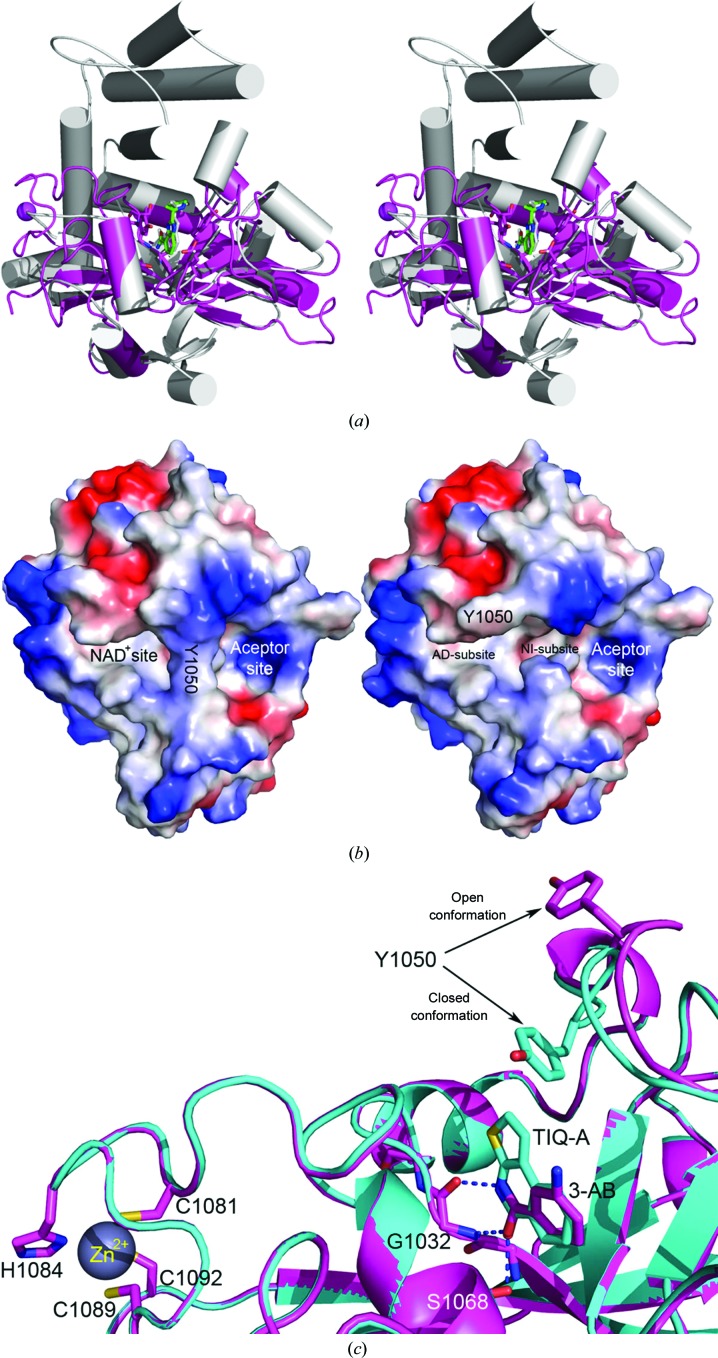
(*a*) Stereoview of the crystal structure of the TNKS2–ABT-888 complex (magenta) super­imposed with the structure of PARP2–ABT-888 (gray; PDB entry 3jkd; Karlberg, Hammarström *et al.*, 2010 ▶). (*b*) The TNKS2 active-site cleft consists of a donor site (NAD^+^ site) and an acceptor site. The left panel illustrates the closed conformation of the TNKS2 PARP domain. The side chain of Tyr1050 from the D-loop divides the acceptor site and the NAD^+^ site. The NAD^+^ site can be further divided into the NI-subsite (where the nicotinamide group is located) and the AD-subsite (which is occupied by the adenosine moiety of NAD^+^). The right panel represents the opened conformation of the TNKS2 PARP domain, in which the side chain of Tyr1050 swings away from the center of the active site and makes the deeply buried NI-subsite widely accessible. (*c*) Close-up view of the NI-subsite of TNKS2 in complex with 3-AB (magenta) and TIQ-A (cyan). The side chain of Tyr1050 from the TNKS2–TIQ-A complex is in the closed confirmation compared with the open conformation of the same residue in the TNKS2–3-AB complex. Also, as shown on the far left of the figure, three conserved cysteine residues and one histidine form a short zinc-binding motif involved in the chelation of Zn^2+^.

The overall crystal structure of the TNKS2 catalytic domain is similar to the structure of the catalytic domain of PARP2 determined in complex with the small-molecule inhibitor ABT-888 (Karlberg, Hammarström *et al.*, 2010 ▶; Fig. 3 ▶ *a*). There are two prominent binding pockets: the NAD^+^ (donor) site and the acceptor site demarcated by the side chain of Tyr1050 in the D-loop in the closed conformation of the catalytic domain (Fig. 3 ▶ *b*, left). Three conserved cysteine residues (*i.e.* Cys1081, Cys1089 and Cys1092) and one histidine (His1084) form a short zinc-binding motif which is unique to TNKS1 and TNKS2 (Fig. 3 ▶ *c*). Two of the inhibitory structures reported here adopt this closed configuration (TNKS2–TIQ-A and TNKS2–BSI-201). The majority of the structures of the catalytic domain bound to inhibitor compounds, however, show an open conformation in which the side chain of Tyr1050 is displaced away from the NAD^+^ site, exposing a narrow and deeply buried pocket for binding the nicotinamide moiety (NI-subsite; Figs. 3 ▶ *b* and 3 ▶ *c*). The residues surrounding this subsite are highly conserved across the whole PARP family. The majority of PARP inhibitors have been designed to target this NI-subsite. A second structural feature of the NAD^+^-binding site is a binding pocket for the adenosine moiety of NAD^+^ (AD-subsite). This subsite encompasses a narrow cleft, which is surface-accessible. The residues surrounding the AD-subsite and the D-loop region are highly conserved in both TNKS and TNKS2, but are distinct compared with other PARP family members. An analysis of the PARP structures deposited in the Protein Data Bank provides little information about the protein–ligand inter­action at the AD-subsite. Our study now provides evidence that the exploitation of ligand interactions at the AD-subsite could improve the design of TNKS2-specific inhibitors.

### 3.1. Group I: inhibitors that only target the NI-subsite (nicotinamide)   

Most of the PARP inhibitors available in the public domain are based on first-generation inhibitors targeting the NI-subsite. In this study, we present eight protein–ligand complex structures that belong to this group. The main interactions between TNKS2 and these inhibitors are (i) hydrogen bonds to the backbone carbonyl and amide group of Gly1032 and the side chain of Ser1068 and (ii) π-stacking interactions between the aromatic ring(s) of the inhibitors with Tyr1060 and Tyr1071. These two specific interactions are observed in the crystal structure complexes of all PARP family members published to date. The half-maximal inhibitory concentrations (IC_50_) measured for these inhibitors with TNKS2 are moderate, ranging from 0.45 µ*M* to over 30 µ*M* (Table 2 ▶), and the values are generally consistent with the binding inter­actions that these inhibitors undergo. The structural details are described below.

#### 3.1.1. 3-Amino-benzamide (3-AB)   

**Figure 4:**
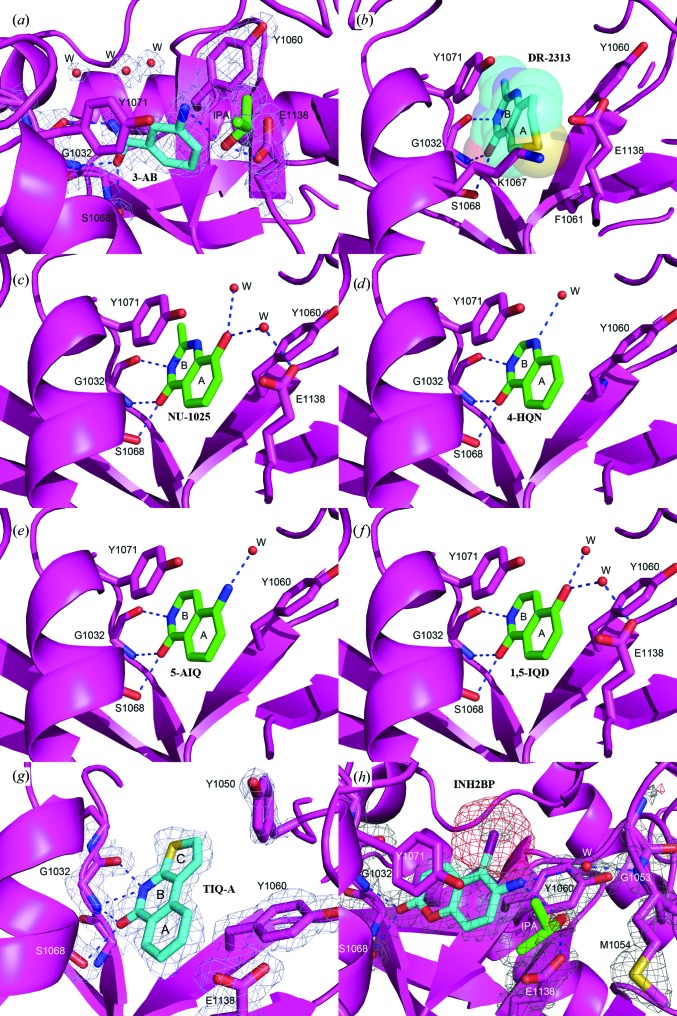
(*a*) The structure of the TNKS2 PARP domain in complex with the first-generation inhibitor 3-AB. 3-AB binds on the bottom of the active site, mimicking the binding mode of nicotinamide. It forms three conserved hydrogen bonds to the backbone of Gly1032 and the side chain of Ser1068. The benzamide ring of 3-AB is in the approximate position to form a π-­stacking interaction with Tyr1071. The 3′ amide of 3-AB forms a connection with the catalytically important residue Glu1138 through a well defined isopropanol molecule (IPA) from the crystallization conditions. The electron density around the inhibitor is a σ-­weighted 2*mF*_o_ − *DF*_c_ map contoured at 1σ. (*b*) The B ring of DR-2313 forms three conserved hydrogen bonds to Gly1032 and Ser1068 and a π-stacking interaction with Tyr1071. The A ring also displays hydrophobic interactions with the catalytically important Glu1138 as well as Tyr1060, Phe1061 and Lys1067. The DR-2313 molecule is represented in a stick form covered by spheres, with the S atom colored yellow. (*c*) NU-1025 forms three hydrogen bonds to Gly1032 and Ser1068 and the π-stacking interaction with Tyr1071 as well as a water-mediated hydrogen bond which links the hydroxyl group of the A ring to Glu1138. (*d*) 4-HQN forms three hydrogen bonds to Gly1032 and Ser1068 and the π-stacking interaction with Tyr1071. (*e*) 5-AIQ has similar interactions with TNKS2: three conserved hydrogen bonds to Gly1032 and Ser1068 and a π-stacking interaction with Tyr1071. (*f*) In addition to the conserved hydrogen bonds and π-stacking interaction, 1,5-IQD makes another water-mediated hydrogen bond from the hydroxyl group of the A ring to Glu1138. (*g*) TIQ-A forms four hydrogen bonds to TNKS2. The B ring forms three hydrogen bonds to the backbone of Gly1032 and one to the side chain of Ser1068. The tricyclic ring of TIQ-A accounts for a larger planar surface and forms a π-stacking interaction with Tyr1071 compared with the other one-ring or two-ring inhibitors from the same inhibitor class. The side chain of Tyr1050 from the D-loop also swings towards TIQ-A and adopts a closed conformation. (*h*) INH_2_BP binds to the NI-subsite differently from the other PARP inhibitors observed in this study. The inhibitor adopts a position in which the iodine moiety points towards the AD-subsite. It does not preserve the three critical hydrogen bonds on the bottom of the NI-subsite observed for 3-AB or TIQ-A. Instead, the hydroxyl group forms only two hydrogen bonds to the main chain of Gly1032 and the side chain of Ser1068.

3-AB is the most studied first-generation PARP inhibitor. It has been co-crystallized with several PARP proteins, including PARP2 (PDB entry 3kcz; Karlberg, Hammarström *et al.*, 2010 ▶), PARP10 (PDB entry 3hkv; Structural Genomics Consortium, unpublished work), PARP12 (PDB entry 2pqf; Structural Genomics Consortium, unpublished work) and PARP14 (PDB entry 3goy; Wahlberg *et al.*, 2012 ▶). We determined the structure of the TNKS2 catalytic domain in complex with 3-AB to 1.9 Å resolution. Similar to the previously reported structures listed above, 3-AB sits on the bottom of the active site, mimicking the binding mode of nicotinamide. It forms three conserved hydrogen bonds to the backbone carbonyl and amide of Gly1032 and the side chain of Ser1068. The benzamide ring of 3-AB (A ring) is in an approximate position to stack with Tyr1071 and Tyr1060. Nevertheless, the 3′-substituted amide group of 3-AB forms a weak hydrogen-bond interaction with the O^η^ atom of Tyr1071 (3.1 Å), which pulls the plane of 3-AB closer to Tyr1071 and away from Tyr1060. It should be noted that the 3′ amide of 3-AB forms a hydrogen bond to a well defined isopropanol molecule (IPA) acquired from the crystallization solution. The alcohol links 3-AB to the catalytically important residue Glu1138 (Fig. 4 ▶ *a*).

#### 3.1.2. 2-Methyl-3,5,7,8-tetrahydro-4*H*-thiopyrano[4,3-*d*]pyrimidin-4-one (DR-2313)   

DR-2313 is a potent, water-soluble competitive PARP inhibitor. It is also the first PARP inhibitor that does not contain a benzamide substructure, which had previously been thought to be essential for good binding to PARP enzymes. In DR-2313, the amide group is fused into the B ring, a modification that improved the binding potency (Nakajima *et al.*, 2005 ▶). The IC_50_ value for DR-2313 from our study is about ten times better than the value for 3-AB (Table 2 ▶). In the publicly available structure of the complex of PARP3 with DR-2313 at 2.1 Å resolution (PDB entry 3c4h; Lehtiö *et al.*, 2009 ▶), the inhibitor adopts a binding mode similar to that seen in our 1.5 Å high-resolution structure (Fig. 4 ▶ *b*). In addition to the three conserved hydrogen bonds associated with the B ring, and the π-stacking with Tyr1071, the bulkier S atom from the A ring also displays hydrophobic interactions with Glu1138, Lys1067, Phe1061 and Tyr1060.

#### 3.1.3. 8-Hydroxy-2-methyl-3-hydro-quinazolin-4-one (NU-1025)   

NU-1025 forms the three hydrogen bonds with Gly1032 and Ser1068 along with the π-stacking interaction with Tyr1071 seen with our other structures described above. In the 2.2 Å resolution electron-density map, a water molecule can be identified which links the hydroxyl group from the A ring to the catalytically important Glu1138. This additional hydrogen bond mediated by the water molecule may contribute to the lower IC_50_ of NU-1025 compared with that of DR-2313 (Table 2 ▶, Fig. 4 ▶ *c*). The second structural water molecule hydrogen-bonded to the hydroxyl group has a very weak interaction with the protein and for this reason contributes very little to the ligand potency increase (The same is true for 4-HQN, 5-AIQ and 1,5-IQD.) These interactions between NU-1025 and TNKS2 are similar to those previously reported for the PARP1–NU-1025 complex (PDB entry 4pax; Ruf *et al.*, 1998 ▶).

#### 3.1.4. 4-Hydroxyquinazoline (4-HQN)   

4-HQN plays a role in modulating the kinase cascades and regulating transcription factors in a rodent septic shock model (Veres *et al.*, 2004 ▶). Based on our 1.65 Å resolution structure, 4-HGN forms three hydrogen bonds and a π-stacking interaction with Tyr1071 of TNKS2. This configuration of interactions is similar to the interaction of other first-generation PARP inhibitors with TNKS2 (Fig. 4 ▶ *d*) and may explain the similar IC_50_ of 4-HQN and 3-AB towards TNKS2 (Table 2 ▶).

#### 3.1.5. 5-Aminoisoquinolinone (5-AIQ)   

5-AIQ is an isoquinolinone derivative and has been reported to have moderating effects on the organ injury and dysfunction caused by haemorrhagic shock (McDonald *et al.*, 2000 ▶). Our 1.9 Å resolution structure identifies the same three conserved hydrogen bonds and a π-stacking interaction as described for the NI-subsite inhibitors (Fig. 4 ▶ *e*). In concert with this observation, the IC_50_ of 5-AIQ towards TNKS2 was similar to those of 3-AB and 4-HQN.

#### 3.1.6. 1,5-Isoquinolinediol (1,5-IQD)   

The 1.6 Å resolution structure shows that 1,5-IQD forms a water-mediated hydrogen bond from the hydroxyl group of the A ring to Glu1138 in addition to the three conserved hydrogen bonds and the π-stacking interaction, thus interacting with TNKS2 in a manner similar to the NU-1025 TNKS2 catalytic domain complex. The hydrogen bond formed between 1,5-IQD and Glu1138 is likely to contribute to the potent IC_50_ of this inhibitor of 1.5 µ*M*, a level similar to that of NU-1025 (Fig. 4 ▶ *f*).

#### 3.1.7. Thieno-[2,3-*c*]-isoquinolin-5-one (TIQ-A)   

The larger planar surface of its tricyclic ring allows TIQ-A to form an extended π-stacking with Tyr1060 and Tyr1071 of the TNKS2 catalytic domain. The B ring forms three hydrogen bonds to the backbone of Gly1032 and one to the side chain of Ser1068 at the bottom of the NI-subsite of TNKS2. The S atom from the C ring also forms a strong van der Waals interaction with the main-chain carbonyl group of Gly1032 (Fig. 4 ▶ *g*). The tricyclic lactam core of TIQ-A appears to be responsible for its tighter binding to TNKS2 when compared with the two-ring inhibitors discussed above. One surprising observation in the 1.7 Å resolution TNKS2–TIQ-A complex structure is that the side chain of Tyr1050, part of the D-loop, protrudes toward TIQ-A and forms a closed conformation. Additional hydrophobic interactions between the side chain of Tyr1050 and the five-membered thiophene C-ring cause this movement of the D-loop, which brings it closer to the NI-subsite compared with the six inhibitors described above. TIQ-A exhibits the best IC_50_ value towards TNKS2 (0.456 µ*M*) compared with other inhibitors from this category (Table 2 ▶).

#### 3.1.8. 5-Iodo-6-amino-1,2-benzopyrone (INH_2_BP)   

INH_2_BP was designed as a noncovalent inhibitor of PARP1 (Bauer *et al.*, 1995 ▶); however, it displays low potency towards other PARP family members. This inhibitor binds to the NI-subsite of TNKS2 in a mode different from that of all the other PARP inhibitors investigated in this study (Fig. 4 ▶ *h*). Owing to the relatively large radius of the I atom, the inhibitor adopts a position with its iodine end pointing towards the AD-subsite. This orientation prevents the molecule from preserving the three critical hydrogen bonds to residues located at the bottom of the NI-subsite. Instead, only two potential hydrogen bonds to TNKS2 can be observed from INH2BP: one to the main-chain amide of Gly1032 and the other to the side chain of Ser1068 (Fig. 4 ▶ *h*). The amino group on the opposite end of the bicyclic ring system forms three water-mediated hydrogen bonds to Tyr1071, Glu1138 and Gly1053 of TNKS2. It should be noted that the discontinuous 2*mF*_o_ − *DF*_c_ density (contoured at 1σ, black) around the iodine group could be caused by Fourier series truncation ripples, since iodine has a relatively large number of electrons and these ripples compete with normal positive density from the nearby C atom and cancel it out. This ripple effect can be observed as strong negative density surrounding the I atom (Fig. 4 ▶ *h*; *mF*_o_ − *DF*_c_ map contoured at −3σ, colored red).

Inhibitory activities measured for the compounds in group I (Table 2 ▶) correlate well with the number of hydrogen bonds that the inhibitor molecules form to the NI-subsite of TNKS2. For example, TIQ-A is able to form four hydrogen bonds to TNKS2 and has an IC_50_ value of 456 n*M*, which is about three times lower than that for NU-1025 (IC_50_ = 1.4 µ*M*), which forms three direct hydrogen bonds. In distinction, INH2BP forms two direct hydrogen bonds to the NI-subsite and has the least potent IC_50_ (>30 µ*M*).

### 3.2. Group II: inhibitors that reach outside the NI-subsite but do not enter the AD-subsite   

To develop more selective and potent inhibitors for individual PARP1 and PARP2, a series of compounds have been synthesized with substitutions designed to extend towards, but not reach, the AD-site and interact with the N-terminal helices within the catalytic domain. Here, we report the crystal structures of TNKS2 in complex with four such inhibitors.

#### 3.2.1. *N*-(6-Oxo-5,6-dihydro-phenanthridin-2-yl)-*N*,*N*-dimethylacetamide (PJ-34)   

**Figure 5:**
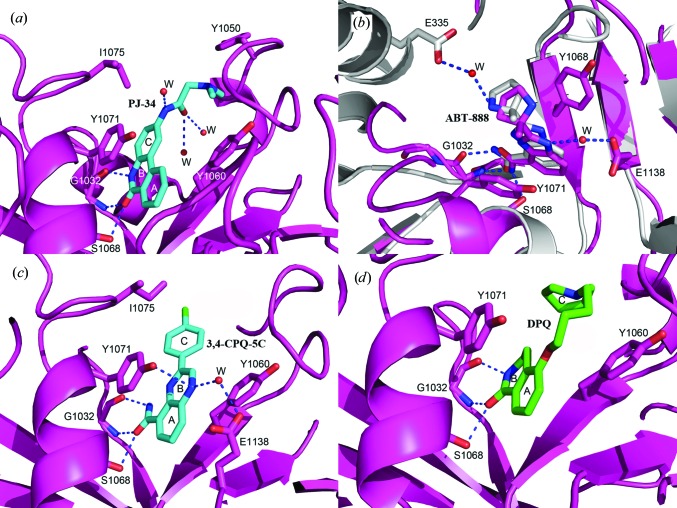
(*a*) The tricyclic lactam core of PJ-34 provides multiple contacts within the NI-subsite, with three conserved hydrogen bonds and extended π-sandwich stacking from Tyr1060 and Tyr1071. Unlike the closed conformation observed in the TIQ-A structure (Fig. 4 ▶ *g*), the tertiary amine extension of PJ-34 pushes the D-loop away from the NAD^+^ site and the side chain of Tyr1050 adopts an open conformation. (*b*) The binding features of ABT-888 with TNKS2 compared with the PARP2 catalytic domains. The pyrrolidine ring of ABT-888 is rotated about 10° towards Glu1138 in the TNKS2 structure compared with its position in the PARP2 complex, with a largest shift of 1.7 Å between the two ABT-888 molecules. At the base of the NI-subsite, the carboxamidyl moiety forms three hydrogen bonds to the backbone of Gly1032 and the side chain of Ser1068 in both TNKS2 and PARP2 (the residue numbering is different in PARP2). The N3 atom of the benzimidazole forms a water-mediated hydrogen bond to Glu1138 in the TNKS2 complex (colored magenta), while the N2 atom of the ABT-888 pyrrolidine forms a water-mediated interaction with a glutamate from the N-terminal helix-bundle domain in the PARP2 complex (colored gray). (*d*) The isoquinolinone base of DPQ contributes to most of the interactions between the inhibitor and TNKS2, with three conserved hydrogen bonds to Gly1032 and Ser1068 as well as π-stacking with Tyr1071 and Tyr1060.

The tricyclic lactam core of PJ-34 creates three conserved hydrogen bonds and an extended π-sandwich stacking with Tyr1060 and Tyr1071 within the NI-subsite of the TNKS2 catalytic domain in a manner similar to TIQ-A. In distinction to the closed conformation observed in the TIQ-A structure, however, the tertiary amine extension protruding from the three-ring core of PJ-34 displaces the D-loop away from the AD-subsite and the side chain of Tyr1050 adopts an open conformation (Fig. 5 ▶ *a*). The amine extension forms two weak water-mediated hydrogen bonds to the backbone of Tyr1050 and Gly1058. The twofold lower IC_50_ of PJ-34 (963 n*M*) compared with TIQ-A is likely to be a result of these distinct interactions with the TNKS2 catalytic domain

#### 3.2.2. 2-[(*R*)-2-Methylpyrol­idin-2-yl]-1*H*-benzimidazole-4-carboxamide (ABT-888, Veliparib)   

A crystal structure of ABT-888 with PARP2 is available in the PDB (PDB entry 3kjd; Karlberg, Hammarström *et al.*, 2010 ▶). In our structure with TNKS2, ABT-888 adopts a similar orientation to that found in the PARP2–ABT-888 complex (Fig. 5 ▶ *b*). At the bottom of the NI-subsite, the carboxamide group forms three hydrogen bonds to the backbone of Gly1032 and the side-chain hydroxyl of Ser1068. The N3 atom of the benzimidazole undergoes water-mediated hydrogen bonding to Glu1138. Fig. 5 ▶(*b*) presents the superposition of ABT-888 complexed to PARP2 and TNKS2; one noticeable difference is the 10° rotation of the pyrrolidine ring of ABT-888 towards Glu1138 in the TNKS2 structure. When complexed with PARP2, the N2 atom of the ABT-888 pyrrolidine forms a water-mediated inter­action with a glutamate from the N-terminal helix-bundle domain of the enzyme. Since this helical structure is absent in TNKS2, the core plane of the ABT-888 scaffold moves towards Glu1138 within the TNKS2 catalytic domain.

#### 3.2.3. 3-(4-Chlorophenyl)-quinoxaline-5-carboxamide (3,4-CPQ-5C)   

As deduced from our 1.57 Å resolution structure, the binding mode of TNKS2 to 3,4-CPQ-5C at the NI-subsite is similar to what has been described for the NU-1025 and 1,5-IQD complexes. The three common hydrogen bonds and the π-stacking interaction lock the carboxamide moiety of the inhibitor tightly into the NI-subsite. Instead of a hydroxyl group, the N9 atom of the quinoxaline ring forms a water-mediated hydrogen bond to Glu1138. The structure of this inhibitor has also been analyzed in complex with PARP1 (PDB entry 1wok; Iwashita *et al.*, 2005 ▶). It was suggested that the terminal phenyl group of this ligand could provide selective inhibition between PARP1 and PARP2. Since TNKS2 lacks the N-terminal helix-bundle domain, 3,4-CPQ-5C adopts a distinct binding mode with the TNKS2 catalytic domain such that the chloro­phenyl group rests in a large pocket adjacent to the NI-subsite and undergoes some hydrophobic interactions with the side chain of Ile1075 (Fig. 5 ▶ *c*).

#### 3.2.4. 3,4-Dihydro-5-[4-(1-piperidinyl)buthoxyl)]-1(2*H*)-iso­quinolinone (DPQ)   

From our 1.8 Å resolution structure of the TNKS2–DPQ complex, we find that the DPQ molecule binds poorly to the protein, with only one of the four active sites (chain *C*) represented in the asymmetric unit displaying electron density sufficient to build in a DPQ model. The isoquinolinone base of DPQ contributes to most of the interactions between the inhibitor and TNKS2, with three conserved hydrogen bonds with Gly1032 and Ser1068 as well as the π-stacking with Tyr1060 and Tyr1071 (Fig. 5 ▶ *d*). As had been found in the 3,4-CPQ-5C complex structure noted above, the extension of the isoquinolinone core does not interact strongly with TNKS2 owing to the absence of the N-terminal helix-bundle domain in the TNKS2 catalytic domain. This feature provides an explanation of why this group of inhibitors in general does not exhibit better selectivity and affinity for TNKS2 compared with PARP1 (as shown in Table 2 ▶).

### 3.3. Group III: inhibitors targeting the AD-subsite (adenosine moiety of NAD^+^)   

#### 3.3.1. 1-Piperazineacetamide-4-[1-(6-amino-9*H*-purin-9-yl)-1-deoxy-d-ribofuranuron]-*N*-(2,3-dihydro-1*H*-isoindol-4-yl)-1-one (EB-47)   

**Figure 6:**
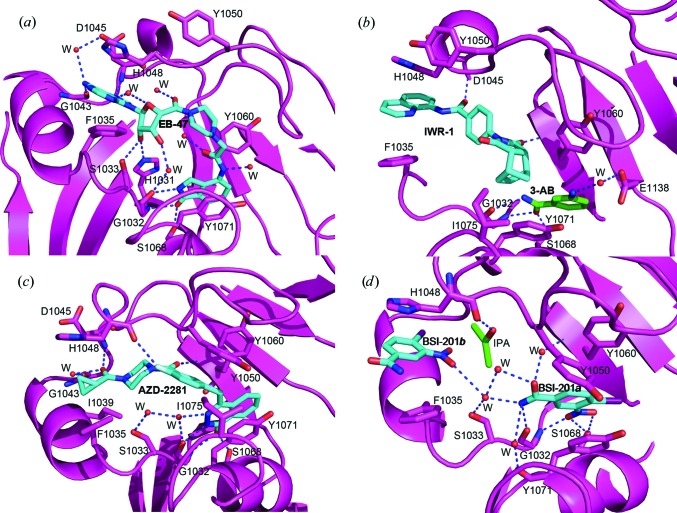
(*a*) EB-47 occupies the entire NAD-binding pocket in a manner that mimics the binding mode of NAD^+^. The isoindolinone core interacts with the three conserved hydrogen bonds and a π-stacking effect of Tyr1060 and Tyr1071 can be observed. The adenosine moiety forms four hydrogen bonds to the surrounding protein residues, including an interaction between the hydroxyl group of the ribose and His1031 within the catalytic core. A network of water-mediated hydrogen bonds further enhances the interactions of the compound within the NAD^+^ donor site. (*b*) In complex with 3-AB, IWR-1 occupies the AD-subsite without perturbation of the NI-subsite. IWR-1 is stabilized in the AD-subsite primarily through hydrophobic interactions. The adenine ring experiences a π-sandwich stacking interaction between Phe1035 and His1048. The other aspect of the IWR-1 ring structure is its orientation into the hydrophobic pocket surrounded by the side chains of Ile1075, Tyr1071 and Tyr1060. Two additional hydrogen bonds are created to Asp1045. 3-AB is present in the co-crystal near the NI-subsite and contributes to the binding stability of the IWR-1 inhibitor and interacts with Tyr1071 through a π-sandwich stacking interaction, a hydrogen bond to Ser1068 and a water-mediated hydrogen bond to Glu1138. (*c*) The bicyclic ring of AZD-2281 binds in the NI-subsite by forming the three critical hydrogen bonds and a π-­sandwich stacking interaction. The fluorobenzyl ring in the central position displaces the D-loop by forming a direct hydrogen bond to the backbone of Phe1048. The carbonyl O atom next to the fluorobenzyl ring forms a direct hydrogen bond to the backbone of Tyr1060. Another carbonyl O atom close to the cyclopropyl ring forms one direct hydrogen bond to the backbone of Asp1045 and one water-mediated hydrogen bond to the backbone of Gly1043. The cyclopropyl ring sits in the AD-subsite in between the aromatic rings of Phe1035 and His1048. (*d*) BSI-201 forms a novel inhibited structure in which two molecules of BSI-201 bind at two distinct sites within the PARP domain of TNKS2. Molecule *A* of BSI-201 is located in the NI-subsite. The nitro group along with the iodine at the 4-position face towards the center of the protein. The nitro group forms three hydrogen bonds, two with Ser1068 and one with Gly1032, mimicking the function of an amide group. The side chains of Lys1067 and Glu1138 adjust themselves to accommodate the nonpolar interaction with the iodine ion. The amide group on the other side of NI-subsite forms a direct hydrogen bond to the main chain of Gly1032. It also contributes to three water-mediated hydrogen bonds to the main chain of Tyr1071, the main chain of Tyr1060 and the side chain of Ser1033. Another observation is that the side chain of Tyr1050 swings towards BSI-201*a* to close the NI-subsite in a manner similar to that observed in the TIQ-A complex structure (Figs. 4 ▶ *a* and 4 ▶ *c*). Tyr1050 also contributes to the hydrophobic environment for BSI-201*a* binding. Molecule *B* of BSI-201 is located in the AD-subsite. The majority of the binding entropy is derived from the π-stacking and nonpolar interactions, with one water-mediated hydrogen bond from the nitro group to the main chain of Asp1045.

EB-47 is an inhibitor that targets not only the NI-subsite but also the AD-subsite within the TNKS2 catalytic domain. The piperazine and succinyl linkers connect the adenosine and isoindolinone cores, making EB-47 one of the most potent TNKS2 inhibitors, with an IC_50_ of 32 n*M*. In the crystal structure, EB-47 occupies the entire NAD-binding pocket, making it an excellent mimic of the NAD-binding mode. The isoindolinone core engages in the well known hydrogen bonds and π-stacking interactions with Tyr1060 and Tyr1071. At the other end, the adenosine moiety forms four hydrogen bonds to surrounding protein residues, with that between the 2′-hydroxyl of the ribose and the catalytically important His1031 seeming to be particularly strong (2.8 Å). In addition, a network of about ten water-mediated hydrogen bonds further locks the compound into the NAD^+^ donor site (Fig. 6 ▶ *a*).

#### 3.3.2. 4-[(3a*R*,4*S*,7*R*,7a*S*)-1,3,3a,4,7,7a-hexahydro-1,3-dioxo-4,7-methano-2*H*-isoindol-2-yl]-*N*-8-quinolinyl-benz­amide (IWR-1)   

This complex structure was obtained by applying the ligand-replacement technique (for details, see §[Sec sec2]2). In two of the four molecules in the asymmetric unit, the NI-subsite still contained a 3-AB molecule which had not been competed out of its binding pocket. As shown in Fig. 6 ▶(*b*), IWR-1 occupies only the AD-subsite not the NI-subsite, owing in part to hydrophobic interactions. A sandwich-like π-stacking interaction with Phe1035 and His1048 holds the adenine-mimicking quinoline ring in position. The substituted isoindole ring binds to the hydrophobic pocket surrounded by the side chains of Ile1075, Tyr1071 and Tyr1060 in the context of 3-AB bound to the NI-subsite. The O atoms of the benzamide group of IWR-1 and of one of the carbonyl substituents of the isoindole substructure form hydrogen bonds to the backbone amides of Tyr1071 and Asp1045, respectively, contributing to the strong binding of this inhibitor. This structure is similar to that recently reported by Narwal *et al.* (2012 ▶) (PDB entry 3ua9), although their complex structure has different crystal packing in space group *C*222_1_.

#### 3.3.3. 4-[3-(4-Cyclopropanecarbonylpiperazine-1-carbonyl)-4-fluorobenzyl]-2*H*-phthalazin-1-one (AZD-2281, Olaparib)   

As shown in Fig. 6 ▶(*c*), the bicyclic ring of AZD-2281 forms the base of the inhibitor that locks into the NI-subsite by forming the three critical hydrogen bonds and the π-sandwich stacking interaction with TNKS2 residues. The central fluorobenzyl ring displaces the D-loop by forming two hydrogen bonds to backbone atoms of Ile1051 and Gly1058 within the TNKS2 catalytic domain. The carbonyl linking the fluorobenzyl ring to the piperazine hydrogen-bonds to the backbone N atom of Tyr1060, while the ketone O atom between piperazine and the cyclopropyl group interacts with the backbone of Asp1045; it also forms a water-mediated hydrogen bond to the backbone amide of Gly1043. The cyclopropyl ring then fits well into the AD-subsite between the aromatic rings of Phe1035 and His1048. AZD-2281 and EB-47 interact with the NI-subsite and the AD-subsite in a similar manner. AZD-2281 may have a more favorable capacity to cross the blood–brain barrier than EB-47 given its lower molecular weight (434.5 *versus* 610.5 Da).

#### 3.3.4. 4-Iodo-3-nitrobenzamide (BSI-201, Iniparib)   

BSI-201 is a noncompetitive PARP1 inhibitor that interacts with the PARP1 zinc-binding site (Buki *et al.*, 1991 ▶; Patel & Kaufmann, 2010 ▶). Mutagenesis studies have confirmed that this benzamide derivative targets and covalently modifies the Arg34 residue in the zinc-finger motif of PARP1 (Melisi *et al.*, 2009 ▶; Ossovskaya & Sherman, 2009 ▶). We included BSI-201 in our analysis in anticipation of obtaining structural information on the TNKS2 PARP domain covalently modified by the BSI-201 adduct. To our surprise, two BSI-201 molecules (BSI-201*a* and BSI-201*b*) bound to the NAD^+^ pocket in two different configurations, with one molecule bound within the NI-subsite and the other in the AD-subsite. The unusual presence of these two BSI-201 molecules was further confirmed by the strong anomalous signal of the I atom of BSI-201 (Fig. 6 ▶ *d*).

BSI-201*a* binds within the NI-subsite. Unlike other benzamide inhibitors, the amide group of BSI-201*a* does not initiate the standard hydrogen bonds that are the basis for the potency of TNKS2 inhibitors. Instead, the nitro group and the iodine linked to the 4-position of the aromatic ring face the center of the protein. The nitro group forms three hydrogen bonds, two with Ser1068 and one with Gly1032, mimicking the interaction pattern of the crucial amide group of other inhibitor molecules. The side chains of Lys1067 and Glu1138 adjust themselves to accommodate the interaction with the I atom. The amide group situated on the opposing side of the aromatic ring forms a hydrogen bond to the main-chain carbonyl of Gly1032. This amide group also forms three water-mediated hydrogen bonds to the main-chain atoms of Tyr1071, the main-chain atoms of Tyr1060 and the side-chain hydroxyl of Ser1033. The side chain of Tyr1050 swings towards BSI-201*a* to cover the NI-subsite, reminiscent of what was observed in the TIQ-A complex (Figs. 3 ▶ *c* and 4 ▶ *g*). Tyr1050 also contributes to the hydrophobic environment for BSI-201*a* binding. The major part of the D-loop moves about 2 Å towards the NAD^+^ donor site when compared with the structures of other complexes (Fig. 3 ▶ *c* and 6 ▶ *a*). This hydrogen-bonding network, together with hydrophobic interactions, holds the inhibitor tightly in the NI-subsite.

A second BSI-201 molecule (BSI-201*b*) is located in the AD-subsite. The majority of its binding energy comes from π-stacking with Phe1035. In addition, BSI-201*b* engages in one water-mediated hydrogen bond from its nitro group to the main chain of Asp1045. In addition to the BSI-201 molecules found in the substrate-binding site, two further BSI-201 molecules are bound to the allosteric site near residue Trp1006 in molecules *A* and *D*. Owing to the crystal packing, this allosteric site in molecules *B* and *C* is occupied by neighboring molecules. The aromatic ring of BSI-201 forms good π-stacking with the side chain of Trp1006.

## 4. Discussion   

In recent years, the members of the PARP family have been the targets of intensive drug-development efforts. To date, more than 40 entries for PARP family members complexed with small-molecule ligands are available in the Protein Data Bank (PDB). Most of these structures were determined in complex with the first-generation PARP inhibitors.

In this report, we have determined 16 novel crystal structures of TNKS2 catalytic domain–inhibitor complexes and highlight several principles of TNKS2 PARP inhibition. The binding modes of the 16 inhibitors have been subdivided into three distinct groups. The first group includes inhibitors that only target the NI-subsite, the second group consists of inhibitors that interact with TNKS2 residues lying outside the NI-subsite but do not contact the AD-subsite, and the third group is represented by inhibitors that target only the AD-subsite. We found that inhibitors that bind to the AD-subsite, such as BSI-201, AZD-2281, IWR-1 and EB-47, dramatically improve the inhibitor potency and are antagonized by movement of the D-loop from the ‘closed’ configuration. An inhibitor that is able to stabilize the D-loop in the closed conformation would be likely to contribute favorably to the energy of binding to TNKS2. We have also determined the high-resolution crystal structures of TIQ-A and BIS-201 complexes, which represent examples of inhibitors that bind to the closed loop conformation. Smaller ligands that interact with the NI-subsite tend to bind to different PARP family members with poor selectivity. Larger ligands that bind to both the NI-subsite and the AD-subsite are good candidates for scaffolds that may demonstrate improved selectivity as TNKS2-specific inhibitors. Although the NAD^+^-binding pockets of tankyrases and other PARP family members are highly conserved, this site may still be exploited to design tankyrase-specific inhibitors. For example, because TNKS and TNKS2 do not have the N-terminal helix-bundle domain located near the AD-subsite of the NAD^+^ pocket in PARP1, which interferes with binding of the dinucleotide, potent inhibitors such as AZD-2281, whose small cyclopropyl group extends into the AD-subsite, demonstrate greater activity against PARP1 than TNKS2. Another approach to target TNKS2 could be to design TNKS2-specific inhibitors based on the AD-subsite structure. Some inhibitors may demonstrate allosteric cooperativity between AD-subsite and NI-subsite binding. For example, IWR-1 may show greater binding and enhanced selective inhibition in the presence of an NI-subsite binder. Lastly, we have determined the crystal structure of the complex of a PARP1 inhibitor that binds to TNKS2 in a completely unique mode. BSI-201 is a potent PARP1 inhibitor that covalently binds and inhibits PARP-1 (Ossovskaya & Sherman, 2009 ▶; Buki *et al.*, 1991 ▶; Melisi *et al.*, 2009 ▶). We observed that BSI-201 bound to TNKS2 in a stoichiometric ratio of 2:1 with the NI-subsites and AD-subsites each bound to one BSI-201 molecule. This mode of binding suggests a new inhibitory mode of noncovalent inhibition of BSI-201 directed towards the TNKS2 catalytic domain.

## 5. Conclusion   

We believe that the high-resolution structural information that we have obtained and systematically analyzed in the context of inhibitor-binding activity experiments will serve as a strong foundation for future tankyrase-specific structure-based drug-discovery programs.

## Supplementary Material

PDB reference: TNKS2–EB-47, 4tk5

PDB reference: TNKS2–DR-2313, 4pnl

PDB reference: TNKS2–3,4-CPQ-5-C, 4tju

PDB reference: TNKS2–BSI-201, 4tki

PDB reference: TNKS2–TIQ-A, 4pnr

PDB reference: TNKS2–5-AIQ, 4pnq

PDB reference: TNKS2–4-HQN, 4pnn

PDB reference: TNKS2–3-AB, 4pml

PDB reference: TNKS2–AZD-2281, 4tkg

PDB reference: TNKS2–NU-1025, 4pnm

PDB reference: TNKS2–PJ-34, 4tjw

PDB reference: TNKS2–INH2BP, 4pns

PDB reference: TNKS2–DPQ, 4tk0

PDB reference: TNKS2–ABT-888, 4tjy

PDB reference: TNKS2–IWR-1, 4tkf

PDB reference: TNKS2–1,5-IQD, 4pnt

## Figures and Tables

**Table:** All 16 complex crystal structures belonged to the orthorhombic space group *P*2_1_2_1_2_1_, with unit-cell parameters of about *a* = 74, *b* = 79, *c* = 153 Å and four molecules in the asymmetric unit. Diffraction data sets were collected using a wavelength of 1 Å. Values in parentheses are for the highest resolution shell.

Inhibitor	3-AB	DR-2313	NU-1025	4-HQN	5-AIQ	1,5-IQD	TIQ-A	INH_2_BP
Data collection								
Resolution (Å)	50.00–1.87 (2.03–1.87)	50.00–1.50 (1.60–1.50)	100.00–2.19 (2.29–2.19)	100.00–1.65 (1.75–1.65)	100.00–1.85 (1.94–1.85)	100.00–1.60 (1.70–1.60)	50.00–1.71 (1.76–1.71)	50.00–1.65 (1.75–1.65)
*R*_merge_ (%)	9.1 (55.8)	5.3 (49.4)	9.9 (53.2)	6.4 (53.4)	8.2 (52.2)	6.2 (52.9)	8.4 (60.6)	6.3 (54.2)
〈*I*/σ(*I*)〉	15.2 (2.3)	18.8 (2.0)	13.4 (3.2)	18.9 (2.2)	16.4 (3.3)	18.0 (2.1)	15.7 (2.7)	17.2 (2.2)
Completeness (%)	95.8 (77.8)	87.4 (58.8)	99.9 (99.3)	96.7 (99.8)	99.9 (99.9)	93.1 (80.9)	97.5 (91.0)	92.4 (81.8)
Multiplicity	6.4 (3.3)	5.7 (2.3)	7.1 (7.1)	6.7 (5.0)	7.3 (7.2)	6.4 (3.8)	7.2 (6.6)	6.4 (3.9)
Refinement								
No. of unique reflections	72800	127552	46616	105727	77647	111457	95691	100927
No. of test-set reflections	1074	1268	1070	1070	990	1120	960	1020
*R*_work_/*R*_free_ (%)	18.0/20.6	18.9/22.8	17.9/22.1	18.6/22.0	18.8/22.7	19.1/22.6	18.2/20.8	18.9/20.9
〈*B*〉 (Å^2^)	33.0	28.7	45.1	29.5	32.2	27.6	28.9	30.3
No. of atoms								
Protein	6525	6643	6555	6523	6550	6575	6501	6456
Ligand	40	48	52	33	36	36	63	39
Zn^2+^	4	4	4	4	4	4	4	4
Water	787	908	407	990	827	982	902	937
R.m.s. deviations								
Bond lengths (Å)	0.010	0.010	0.010	0.010	0.010	0.009	0.010	0.009
Bond angles (°)	1.03	1.07	1.11	1.02	1.02	1.03	1.01	1.01
Ramachandran plot								
Favored (%)	99.37	99.51	97.77	98.12	98.48	98.39	98.61	98.35
Allowed†(%)	0.63	0.49	2.23	1.88	1.52	1.61	1.39	1.65
PDB code	4pml	4pnl	4pnm	4pnn	4pnq	4pnt	4pnr	4pns

**Table:** 

Inhibitor	PJ-34	ABT-888	3,4-CPQ-5C	DPQ	EB-47	IWR-1	AZD-2281	BSI-201
Data processing								
Resolution (Å)	100.00–1.70 (1.80–1.70)	100.00–1.90 (2.00–1.90)	100.00–1.57 (1.66–1.57)	100.00–1.65 (1.75–1.65)	50.00–2.02 (2.12–2.02)	20.00–2.40 (2.49–2.40)	50.00–1.95 (2.05–1.95)	20.00–2.15 (2.25–2.15)
*R*_merge_ (%)	4.9 (41.3)	7.8 (43.2)	5.6 (53.5)	6.2 (53.3)	7.5 (40.3)	12.5 (65.2)	6.8 (56.0)	12.2 (51.2)
〈*I*/σ(*I*)〉	23.7 (2.3)	23.6 (4.2)	19.9 (2.0)	18.5 (2.4)	26.4 (5.2)	14.5 (2.7)	15.0 (2.6)	14.3 (2.4)
Completeness (%)	83.2 (39.4)	98.6 (90.0)	92.1 (77.8)	92.4 (90.5)	99.5 (96.5)	99.6 (99.0)	99.9 (99.0)	99.1 (94.2)
Multiplicity	5.3 (1.4)	13.3 (7.5)	6.2 (3.3)	6.5 (4.9)	14.0 (8.7)	7.2 (7.1)	6.7 (6.8)	6.8 (4.3)
Refinement								
No. of reflections	82691	71110	116789	100882	60027	35340	66638	49502
No. of test-set reflections	1003	1047	1167	997	980	1105	999	1141
*R*_work_/*R*_free_ (%)	17.5/20.5	17.8/21.9	19.4/22.1	18.8/22.5	17.7/21.2	18.6/23.9	18.6/21.6	18.0/22.8
〈*B*〉 (Å^2^)	29.9	32.6	26.4	31.1	34.5	42.2	44.1	29.4
No. of atoms								
Protein	6519	6463	6410	6503	6540	6456	6531	6511
Ligand	66	102	80	144	264	124	128	130
Zn^2+^	4	4	4	4	4	4	4	4
Water	894	799	987	932	802	345	540	553
R.m.s. deviations								
Bond lengths (Å)	0.010	0.010	0.010	0.010	0.009	0.010	0.010	0.010
Bond angles (°)	1.01	1.03	1.07	1.06	1.05	1.15	1.07	1.13
Ramachandran plot								
Favoured (%)	98.51	99.12	98.50	97.99	99.00	97.85	97.65	97.86
Allowed (%)	1.49	0.88	1.50	2.01	1.00	2.15	2.35	2.14
PDB code	4tjw	4tjy	4tju	4tk0	4tk5	4tkf	4tkg	4tki

† No residues were observed in disallowed regions.

**Table 2:** The half-maximal inhibitory concentration (IC_50_) of various inhibitors on TNKS-2 from this study compared with the IC_50_ values available from the literature for various PARPs

			IC_50_ (µ*M*)				
Compound		Chemical structure	TNKS2†	PARP1	PARP2	TNKS1	TNKS2
Group I: inhibitors that only target the NI-subsite							

3-AB	§[Sec sec3.1.1]3.1.1	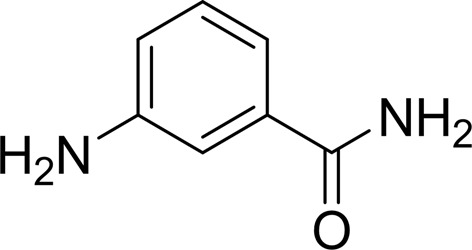	>30	33	—	—	—
DR-2313	§[Sec sec3.1.2]3.1.2	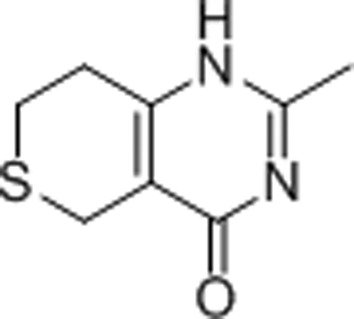	3.0	0.2	0.24	—	—
NU-1025	§[Sec sec3.1.3]3.1.3	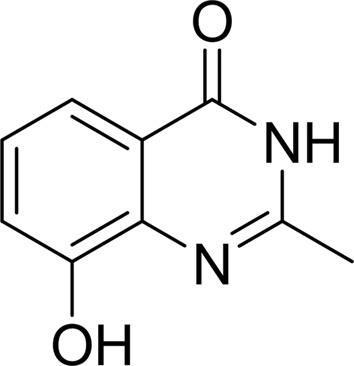	1.4	0.4	—	—	—
4-HQN	§[Sec sec3.1.4]3.1.4	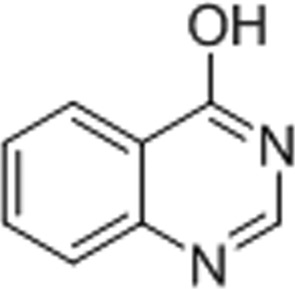	>30	9.5	—	—	—
5-AIQ	§[Sec sec3.1.5]3.1.5	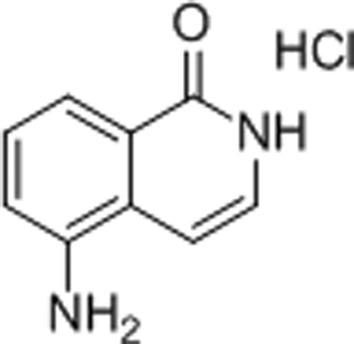	10	0.25	—	—	—
1,5-IQD	§[Sec sec3.1.6]3.1.6	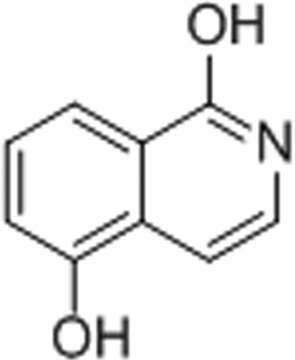	1.5	0.39	—	—	—
TIQ-A	§[Sec sec3.1.7]3.1.7	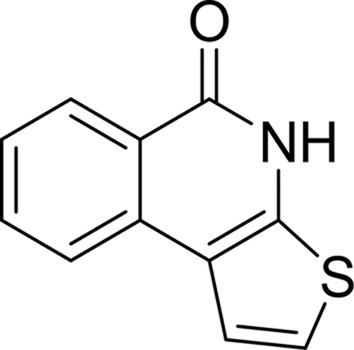	0.456	0.45	—	—	—
INH_2_BP	§[Sec sec3.1.8]3.1.8	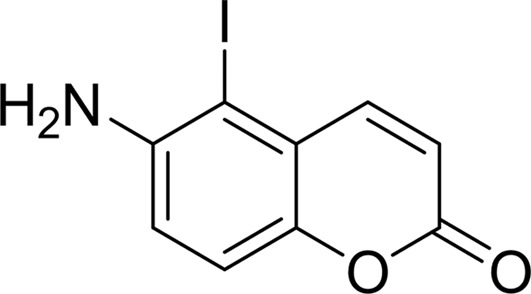	>30	5.07	4.75	—	—
Group II: inhibitors that reach outside the NI-subsite but do not enter the AD-subsite							

P-J34	§[Sec sec3.2.1]3.2.1	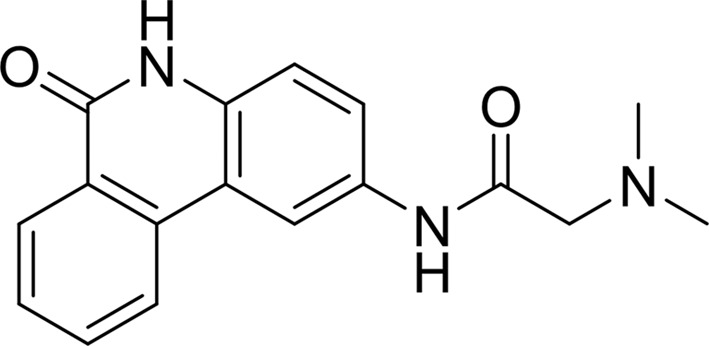	0.963	0.02	—	—	—
ABT-888	§[Sec sec3.2.2]3.2.2	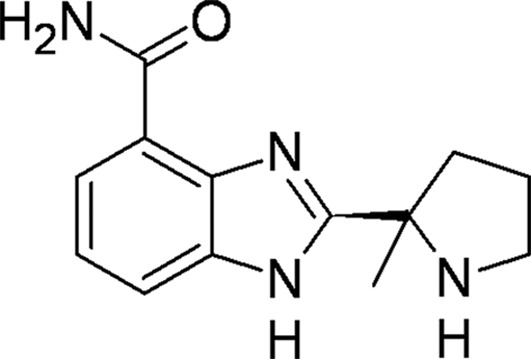	0.367	0.008	0.011	14.97	6.52
3,4-CPQ-5C	§[Sec sec3.2.3]3.2.3	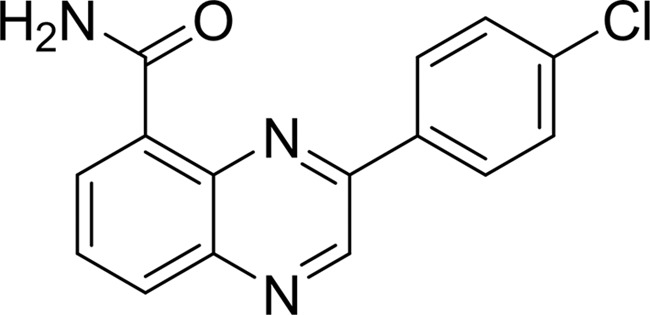	3.6	—	—	—	—
DPQ	§[Sec sec3.2.4]3.2.4	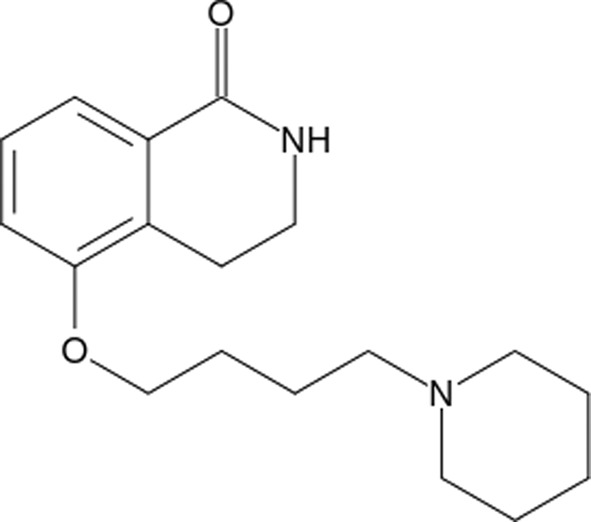	2.8	0.023	—	0.033	—
Group III: inhibitors targeting both the NI-subsite and AD-subsite							

EB-47	§[Sec sec3.3.1]3.3.1	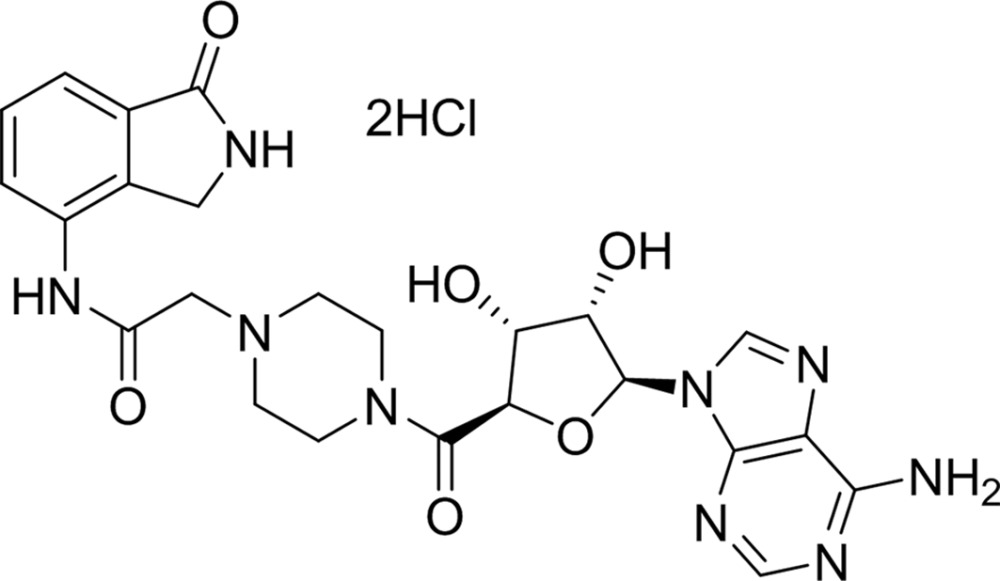	0.032	—	—	—	—
IWR-1	§[Sec sec3.3.2]3.3.2	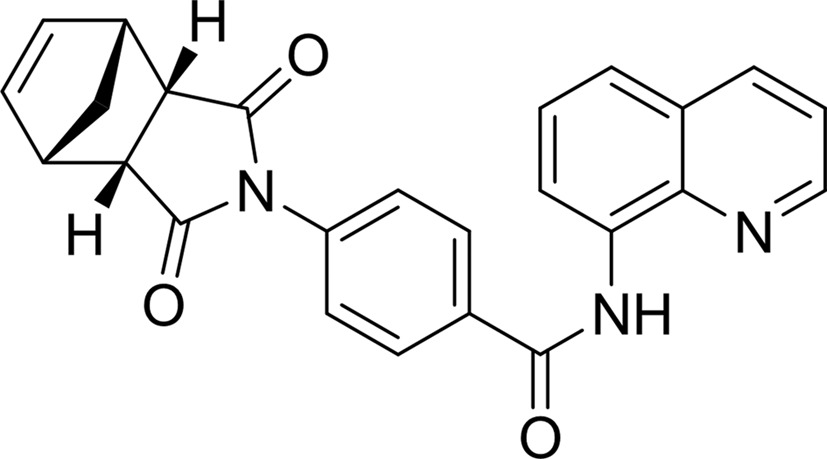	0.285	>18.75	>18.75	0.1–1.9	0.056–0.78
AZD-2281	§[Sec sec3.3.3]3.3.3	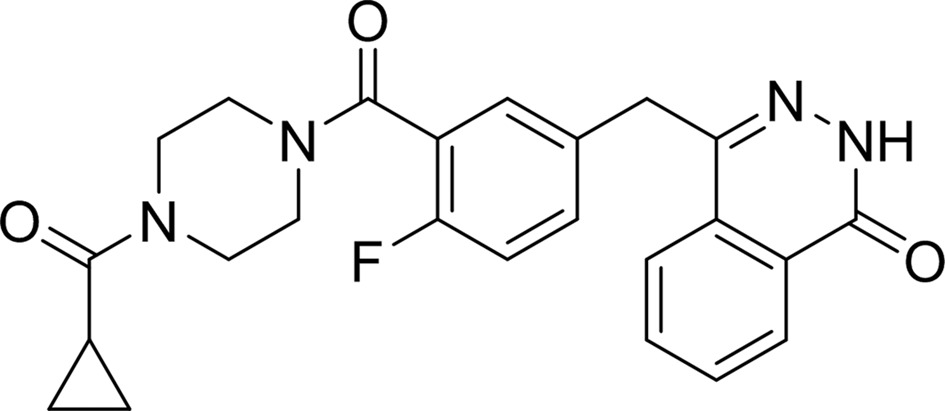	0.140	0.005	—	—	—
BSI-201	§[Sec sec3.3.4]3.3.4	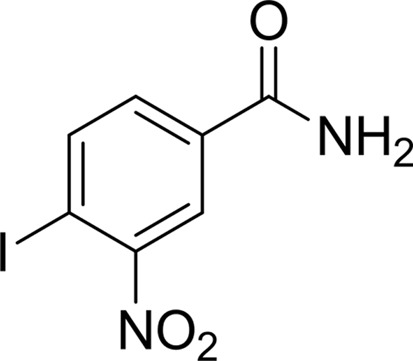	0.416	—	—	—	—

† Values from this study.
